# Follow-up of humoral and cellular immune responses after the third SARS-CoV-2 vaccine dose in multiple myeloma patients

**DOI:** 10.3389/fimmu.2025.1532947

**Published:** 2025-02-18

**Authors:** Vincenzo Raimondi, Paola Storti, Rosanna Vescovini, Valentina Franceschi, Denise Toscani, Laura Notarfranchi, Anna Benedetta Dalla Palma, Nicolas Thomas Iannozzi, Sergio Minesso, Matteo Scita, Oxana Lungu, Mattia Dessena, Gaetano Donofrio, Nicola Giuliani

**Affiliations:** ^1^ Department of Medicine and Surgery, University of Parma, Parma, Italy; ^2^ Department of Medical-Veterinary Science, University of Parma, Parma, Italy; ^3^ Hematology, “Azienda Ospedaliero-Universitaria di Parma”, Parma, Italy

**Keywords:** multiple myeloma, SARS-CoV-2 vaccination, humoral immunity, T cell response, Omicron variant, breakthrough infection

## Abstract

The stability of immune responses to SARS-CoV-2 vaccines, especially concerning the cross-reactive recognition of the Omicron variant, remains incompletely characterized in multiple myeloma (MM) patients. This study evaluated humoral responses in 29 MM patients and cellular responses in a subset of 19 MM patients, specific to Wuhan and Omicron spike proteins, between 16 and 26 weeks following the third vaccine dose. After 26 weeks, we highlighted a significant reduction in the neutralizing antibodies to both spikes and the percentages of IFN-γ^+^CD107a^+^ spike-specific CD8^+^ T cells. On the other hand, patients who underwent an additional stimulation between the two time points, through either a fourth vaccine dose or breakthrough infection, showed a significant increase in neutralizing antibodies and stable levels of cytotoxic CD8^+^ T cells. Additionally, those with only three doses experienced a higher rate of breakthrough infections during the 32-week follow-up period. These findings underscore the waning of vaccine-induced immunity over time and may help benefit-risk evaluation in vaccination strategies in MM patients.

## 1 Introduction

Multiple myeloma (MM) is a hematological malignancy associated with severe impairments of humoral and cellular immune responses (1). These deficits increase the susceptibility of MM patients to severe infections, including those caused by SARS-CoV-2 (2, 3). Due to the inherent immunodeficiency associated with the disease and the immunosuppressive nature of anti-MM therapies, patients with MM have shown elevated rates of COVID-19-related morbidity and mortality, with mortality rates reaching up to 33% at the pandemic onset (4). Emerging data suggest that clinical outcomes, particularly in terms of overall survival (OS), have shown an improvement across the various viral phases of the pandemic, likely reflecting both advances in disease management and the protective benefits conferred by successive vaccine doses (5).

Vaccination against SARS-CoV-2 represents a cornerstone in preventing COVID-19 in immunocompromised patients, including those with MM, smoldering MM, or monoclonal gammopathy of undetermined significance (2, 6, 7). While vaccines authorized in the European Union, such as mRNA-based (BNT162b2, mRNA-1273) and vector-based (ChAdOx1-S, Ad26.COV2.S) vaccines, have demonstrated high efficacy in the general population, eliciting up to 95% protection, the immune response in MM patients has been markedly compromised (8, 9). Multiple studies have reported inconsistent seroconversion rates, influenced by variables such as patient demographics, disease status, and ongoing treatments (10–19).

In response to the diminished immunogenicity, a third dose of mRNA vaccines has been recommended for immunocompromised individuals, including MM patients, to restore and augment the immune defenses against the SARS-CoV-2 variants of concern (VoC) that emerged during the pandemic waves (18, 20). Our previous study demonstrated that the booster dose (the third vaccine dose) led to a robust increase in neutralizing antibody titers against nearly all variants tested in newly diagnosed MM patients (MMD). However, in relapsed/refractory patients (MMR), the Omicron variant retained a detrimental impact on neutralizing capacity, highlighting the need for optimized vaccination strategies in this subgroup (18). Moreover, although Ntanasis-Stathopoulos et al. (21) demonstrated that a fourth dose of the BNT162b2 vaccine significantly enhanced the humoral response in patients with MM, Hofsink et al. (22), in the COBRA KAI cohort study, observed that, despite a marked improvement, the levels of spike-IgG antibodies following the fourth mRNA vaccination remained significantly lower compared to those observed in age-matched healthy individuals after their third dose.

Finally, despite the protective effect of additional SARS-CoV-2 vaccine doses in immunocompromised individuals, concerns remain about the strength and waning of vaccine-induced immunity, particularly in high-risk populations (23–26). Tut et al. (27) demonstrated that, although the administration of a third dose in elderly care home residents (one of the most vulnerable population groups) elicits a significant increase in antibody levels, these titers can decline by 21-78% within 100 days post-vaccination, with breakthrough infections occurring in up to 27% of individuals, primarily due to immune-evasive variants such as Omicron (27). Concerning MM patients, limited long-term data suggest a similar trend, with neutralizing responses decreasing over time, particularly in the absence of additional immune stimulation (28).

The impairment of humoral immunity in these patients is further exacerbated by defective CD8^+^ T cell responses following vaccination, which may impair long-term protection, especially against variants with immune escape capabilities like Omicron subvariants (29). In line with this, the European Myeloma Network has highlighted the importance of updated mRNA-based vaccines for MM patients, as well as continued preventive measures to mitigate COVID-19 risk (30). These observations underscore the critical need for continuous monitoring of the durability of immune protection and for strategies to sustain robust immunity in high-risk populations, such as MM patients.

In the present study, we conducted a prospective observational study aimed at evaluating the stability of both humoral and cellular immune responses specific to the Wuhan and Omicron variant spike proteins in a real-world cohort of MM patients, between 16 and 26 weeks following the third SARS-CoV-2 vaccine dose. During the study, some patients experienced an additional immune stimulation, either through a breakthrough infection or a fourth vaccine dose. Consequently, we also examined how this additional stimulation affected spike-specific immune responses and the incidence of new infections. Moreover, MM patients were stratified based on key demographic and clinical characteristics, including age, disease status (MMD vs MMR), and ongoing treatments such as immunomodulatory agents (IMiDs) and steroids, allowing for a detailed assessment of their potential impact on immune responses.

## 2 Materials and methods

### 2.1 Study design and participants

All patients were followed and treated at the Hematology Unit of Parma Hospital and received vaccinations as part of the national SARS-CoV-2 vaccination program. From February 2022 to March 2022, 29 consecutive patients with MM were enrolled in the study, comprising 14 patients with MMD and 15 with MMR. Each patient has completed the vaccination cycle consisting of three doses of mRNA SARS-CoV-2 vaccine and was followed until September 2022.

**Figure 1:**
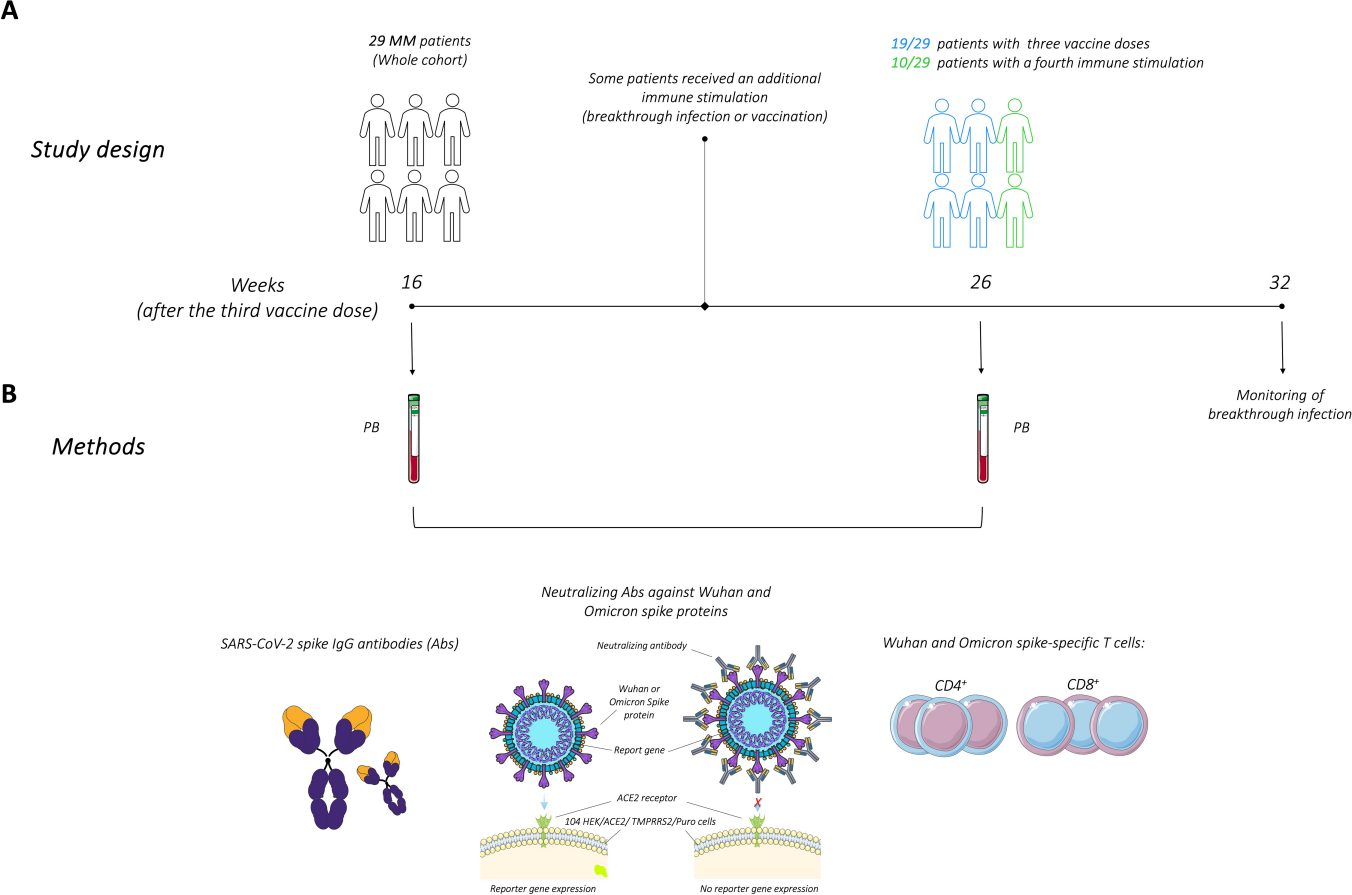
Study design and methods for evaluating immune responses following the third SARS-CoV-2 vaccine dose in multiple myeloma (MM) patients. **(A)** Study design illustrating patient’s cohort and time points of peripheral blood (PB) collection, at 16 and 26 weeks after the third dose of the SARS-CoV-2 vaccine. Some patients underwent additional immune stimulation between these two time points. Monitoring of breakthrough infections was conducted for up to 32 weeks. **(B)** SARS-CoV-2 spike IgG antibodies, neutralizing antibodies along with CD4^+^ and CD8^+^ T cells responses specific to the Wuhan and Omicron spike proteins, were analyzed. The figure was generated from adapted figures provided by Servier Medical Art (Servier; https://smart.servier.com/), licensed under a Creative Commons Attribution 4.0 Unported License.

Peripheral blood (PB) samples were collected at two distinct time points during the study: at 16 weeks (median 114 days, range 85-124) and 26 weeks (median 182 days, range 162-207) after the administration of the third vaccine dose (21 patients received mRNA-1273; 8 patients received BNT162b2). Notably, all MM patients had previously received the BNT162b2 vaccine for both the first and second doses. The monitoring of breakthrough infection, confirmed by PCR or antigen swab test, was conducted until 32 weeks (median 334 days, range 272-340) after the third vaccine dose. During the study conduction, the fourth SARS-CoV-2 vaccine dose became available through the national booster administration program, starting for residents in Emilia Romagna region on April 13^th^, 2022. Between the two PB samples, 10 patients (37% of participants) underwent a fourth immune stimulation: 7 patients chose to receive a fourth vaccine dose (BNT162b2), while 3 patients experienced a breakthrough infection. The whole cohort was initially analyzed to assess the overall stability of the immune response, with subsequent analyses stratified into two groups based on the number of immune stimulations. The study design, time points of PB collection, and methods are illustrated in **Figure 1**.

### 2.2 Ethics statement

PB samples were obtained according to the criteria of the Declaration of Helsinki and following written informed consent. The study was approved by the Local Ethics Committee.

### 2.3 Detection of SARS-CoV-2-specific IgG antibodies

Heat-inactivated sera samples were tested for SARS-CoV-2-specific IgG antibodies using a commercial quantitative two-step ELISA (COVID-SeroIndex, Kantaro Quantitative SARS-CoV-2 IgG Antibody Kit, R&D Systems, Minneapolis, MN, USA), according to the manufacturer’s recommendations. Quantitative results were reported in Binding Antibody Unit per milliliter (BAU/mL) calculated by multiplying the Kantaro ELISA value (Arbitrary Unit per milliliter, AU/mL) by the conversion factor 0.007 (derived from the NIBSC Anti-SARS-CoV-2 Antibody Diagnostic Calibrant [NIBSC code 20/162]) and by the dilution factor of 200. The assay’s lower limit of detection was at <4.48 (BAU/mL).

### 2.4 SARS-CoV-2 pseudoviruses generation and neutralization assay against the original viral strain and Omicron variant

Lentiviral vector-based SARS-CoV-2 spike pseudoviruses were generated as previously described (18, 31). SARS-CoV-2 spike pseudoviruses displayed on their surface 2 different spike glycoproteins: Wuhan original strain (B.1 Lineage; China) or Omicron (B.1.1.529 Lineage; Europe), the locally dominant variant at the time of the study.

The neutralization assay was performed on 104 HEK/ACE2/TMPRRS2/Puro cells (31) testing heat-inactivated sera samples at the dilution of 1:4–1:8–1:16–1:32–1:64–1:128–1:256–1:512–1:1024 as previously described (18). A negative control was established without serum. The relative luciferase units (RLUs) were compared and normalized to those derived from wells where pseudovirus was added in the absence of sera (100%). Neutralization titer 50 (NT_50_) was expressed as the maximal dilution of the sera where the reduction of the signal was ≥50%. Notably, NT_50_ values were multiplied by 40 to account for the initial serum volume of 0.025 mL, ensuring normalization to a standard volume of 1 mL (31). The neutralization titers’ lower detection limit was 160 NT_50_/mL. Each serum was tested in triplicate.

### 2.5 Intracellular cytokine staining flow cytometry T cell assay

PB mononuclear cells (PBMCs) were isolated from patients’ blood samples by density gradient centrifugation. The cells were resuspended in a complete RPMI medium supplemented with 10% heat-inactivated calf serum (Biochrom, GmbH), 2 mM L-glutamine, and 1% penicillin-streptomycin, followed by counting and cryopreservation in cold freezing medium. PBMCs collected at 16 and 26 weeks from each patient were thawed simultaneously, resuspended in a complete medium, and incubated at 37°C for 6 hours to allow resting. Following this, 1 × 10^6^ PBMCs per tube from each sample were incubated with CD107a (cat. 555801, BD Pharmingen, Franklin Lakes, NJ, USA), monensin (cat.554724, BD Golgi Stop, BD Biosciences), and S1 and S2 peptide pools covering Wuhan spike protein or O1 and O2 peptide pools covering Omicron spike protein (PepMIX SARS-CoV-2 spike glycoprotein, cat. PM-WCPV-S-3, and PepMix SARS-CoV-2 spike BA.1/Omicron, cat.PM-SARS2-SMUT08-1, JPT Peptide Technologies GmbH, Berlin, Germany) added at a final concentration of 1 μg/mL. For each tested sample, a positive control (*S. enterotoxin B* at 2 μg/mL, Merck KGaA, Darmstadt, Deutschland) and an unstimulated control (stimulation with an equimolar amount of DMSO, Merck KGaA) were also included. After 2h of incubation, brefeldin A (5 μg/mL, Merck KGaA) was added and the samples were incubated for an additional 16h. PBMCs were washed and stained with BD Horizon Fixable Viability stain 575 V (1:1000). A surface staining cocktail was added containing saturating concentrations of BV480 CD3 (cat.566105, BD Horizon), BV786 CD4 (cat.563877, BD Horizon) and BV711 CD8 (cat.563677, BD Horizon). PBMCs were fixed and permeabilized using FACS Lysing Solution 1x and FACS Permeabilizing Solution 2 1x (cat.349202 and cat. 340973, BD Biosciences). After washes, PBMCs were stained with a cocktail of anti-human IFN-γ-FITC (cat.554700, BD Pharmingen), IL-2-PerCP-Cy5.5 (cat.560708, BD Pharmingen), and TNF-α-BV421 (cat.562783, BD Horizon). At least 0.75 × 10^6^ total events per sample were acquired using a BD FACSCelesta flow cytometer (BD Bioscience) operated with FACSDiva Software (version 8.02, BD Bioscience). Single-fluorochrome compensation was performed using BD CompBeads (cat. 552843) and PBMCs. A hierarchical gating strategy, established during assay qualification, was consistently applied to all sample analyses, as previously described (18). Peptide-specific responses were calculated by subtracting the values of unstimulated controls from their corresponding peptide-stimulated samples. Data are expressed as the percentage of total CD4^+^ or CD8^+^ T cells, with the lower limit of detection set at <0.001 of the parental gate.

### 2.6 Statistical analysis

Data are presented as individual data points with lines connecting paired samples where applicable. Bars represent the median with interquartile ranges (IQRs). Paired samples were analyzed using the Wilcoxon test, while unpaired samples were analyzed using the Mann–Whitney U test. Correlation coefficients were calculated using the Spearman rank correlation method. All statistical tests were two-sided, with a nominal significance threshold set at *P* < 0.05 unless otherwise specified. Data analysis and graphical representations were performed using GraphPad Prism software, version 10.1.1. Statistical significance is indicated as follows: *P < 0.05, **P < 0.01, ***P < 0.001, ****P < 0.0001.

## 3 Results

### 3.1 Patient characteristics

The study cohort included 29 patients with MM, comprising 14 patients with MMD and 15 with MMR. The cohort’s median age was 77 years (range 51-86), with a predominance of males (55.2%, 16 out of 29). The median infiltration of bone marrow plasma cells (PCs) was 35% (range 2-98%). All MMD patients had received first-line treatment, whereas patients with MMR had received at least two lines of treatment (range 2-6). The characteristics of the whole cohort of patients included in the study are shown in **Supplementary Table 1**.

### 3.2 Stability of humoral responses after the third vaccine dose in MM patients

**Figure 2:**
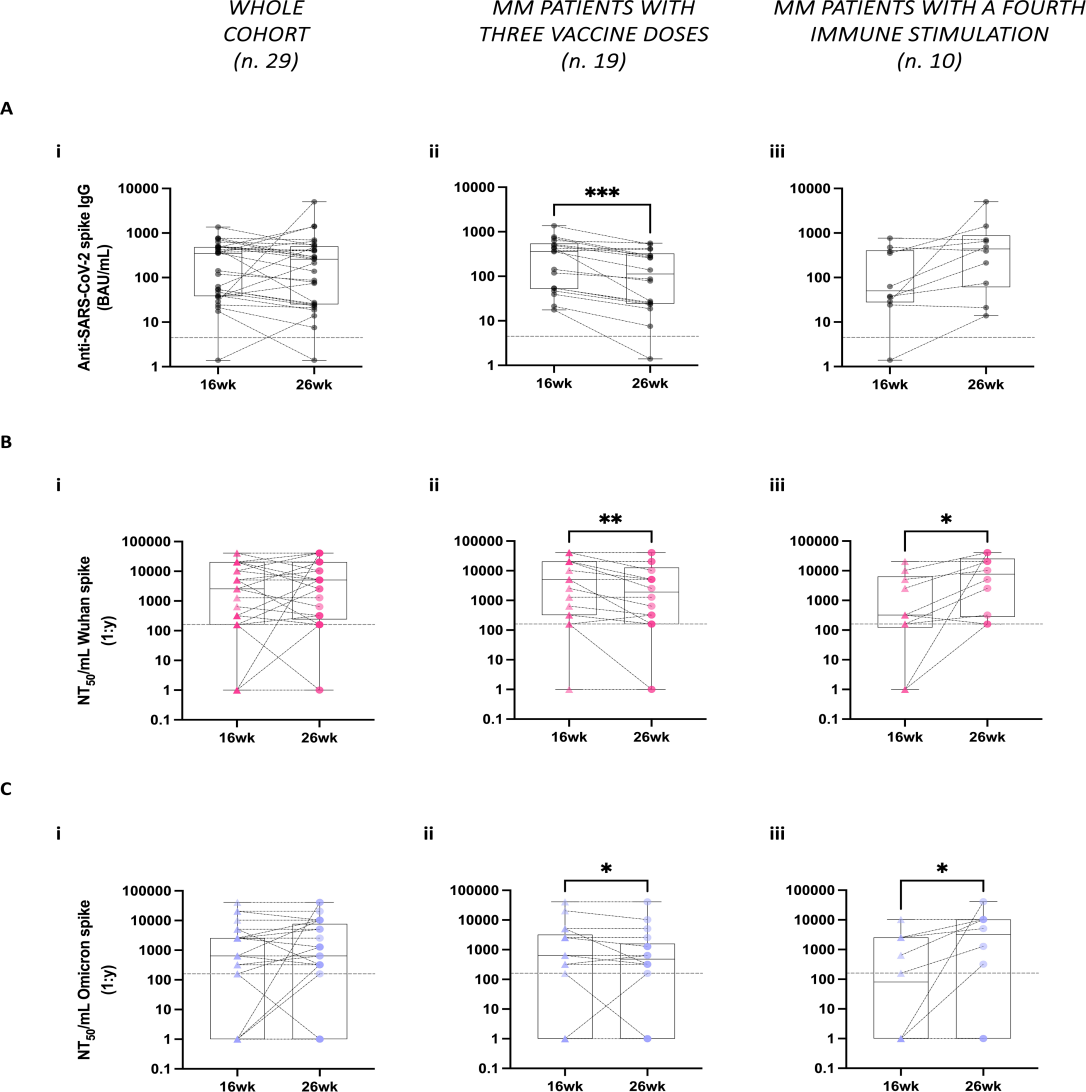
Stability of humoral responses against SARS-CoV-2 following third vaccine doses spike in multiple myeloma (MM) patients. **(A)** Spike-specific IgG antibody levels were analyzed at 16- and 26-weeks (wk) post-administration of the third vaccine dose across the whole MM patient’s cohort **(Ai)**, in the subgroup of MM patients who received only three vaccine doses **(Aii)**, and in the subgroup of MM patients who underwent additional immune stimulation between the two time points **(Aiii)**. Spike-specific IgG antibodies were measured using the COVID-SeroIndex Kantaro SARS-CoV-2 IgG test. Quantitative results are reported in Binding Antibody Unit/mL (BAU/mL). **(B, C)** Neutralizing antibody titers against both the Wuhan **(B)** and the Omicron spike proteins **(C)** were analyzed in the whole cohort **(Bi, Ci)**, as well as separately in patients with only 3 vaccine doses **(Bii, Cii)** and those with additional fourth immune stimulation **(Biii, Ciii)**. Quantitative results are reported in Neutralization titer 50/mL (NT_50_/mL) as the maximal dilution of the sera where the reduction of the signal is ≥50%. In **A–C**, the limit of detection is indicated by the horizontal dotted line. Individual data points are shown, lines connect paired samples, and bars show the median with IQRs. Statistical analysis was performed using a paired, two-tailed, non-parametric Wilcoxon test, with *P* values reported when *P* < 0.05 (*P < 0.05, **P < 0.01, ***P < 0.001).

We evaluated spike-IgG antibody levels between 16 and 26 weeks following the administration of the third vaccine dose in our cohort of MM patients. We found that at 16 weeks, the seropositivity (detectable levels of spike-IgG antibodies in the patient’s sera) rate in the whole cohort was 96.5% (28 out of 29) with only one patient exhibiting no detectable antibodies. Across the whole cohort, between the two time points at 16 weeks and at 26 weeks after the third vaccine dose, no significant differences were observed in the spike-IgG antibody levels (**Figure 2Ai**). However, a significant decrease in spike-IgG antibody levels was noted in the subgroup of patients who received only the three-dose vaccination regimen (**Figure 2Aii**). In contrast, the patients who underwent a fourth stimulus, either through breakthrough infection or a fourth vaccine dose, did not show a significant decrease in spike-IgG antibody levels over time (**Figure 2Aiii**).

To examine the functional quality of these vaccine-induced antibodies, we tested patients’ sera using the neutralization assay against two pseudoviruses displaying Wuhan or Omicron spike proteins on their surface. Similar to total IgG levels, no significant differences were observed in the neutralizing antibody (nAb) responses to both Wuhan and Omicron variant spikes between the two time points across the whole cohort (**Figures 2Bi, 2Ci**). However, in the subgroup of patients who received only the three vaccine doses, there was a significant reduction in nAb titers between the two time points against the two spike variants analyzed (**Figures 2Bii, 2Cii**). In contrast, patients who underwent a fourth stimulation exhibited a significant increase in nAb titers within the same period in response to both spike proteins (**Figures 2Biii, 2Ciii**).

Additionally, we analyzed the correlation between spike-specific IgG antibody levels and nAb titers against the Wuhan and Omicron spike proteins. As expected, significant correlations were observed at both time points across the whole cohort (**Supplementary Figures 1Ai, 1Aii, 1Bi, 1Bii**, respectively).

To explore potential confounding factors influencing humoral responses, patients were stratified by age (<77 vs ≥77 years, based on the median age) and disease status (MMD vs MMR). Among patients who received the three-dose vaccination regimen, no statistically significant differences in spike-specific IgG levels or nAb titers were detected (**Supplementary Figures 2A–C**). In contrast, significant differences were observed in patients who received a fourth immune stimulation. At 26 weeks, patients aged ≥77 years exhibited significantly lower spike-specific IgG levels and nAb titers against both the Wuhan and Omicron spike proteins compared to those aged <77 years (**Supplementary Figures 2Di, 2Ei, 2Fi**). Similarly, patients with MMR displayed significantly lower spike-specific IgG levels at 26 weeks compared to patients with MMD (**Supplementary Figure 2Dii**). However, no significant differences in nAb titers were observed between MMR and MMD patients (**Supplementary Figures 2Eii, Fii**).

Finally, we evaluated the potential impact of anti-myeloma therapies, specifically IMiDs and steroids, on humoral responses. No significant differences were observed in patients undergoing these treatments (data not shown).

Overall, these findings demonstrate a reduction in neutralization capacity against both the Wuhan original strain and the Omicron variant between 16 and 26 weeks following a three-dose vaccination regimen. Additional immune stimulations, whether through natural infection or a booster dose, appear to contribute significantly to the maintenance or enhancement of both anti-SARS-CoV-2 IgG serum levels and nAbs capacity, including against VoC. However, older patients and those with MMR exhibited less efficient responses to these additional stimulations.

### 3.3 Stability of T cell responses after the third vaccine dose in MM patients

The coordinated activation of both humoral and cellular immune responses plays a crucial role in protection against SARS-CoV-2 infection. While SARS-CoV-2 vaccines aim to elicit durable immunological memory encompassing both nAbs and virus-specific T cells, the stability and cross-reactivity of T cell responses in MM patients remain insufficiently characterized.

**Figure 3:**
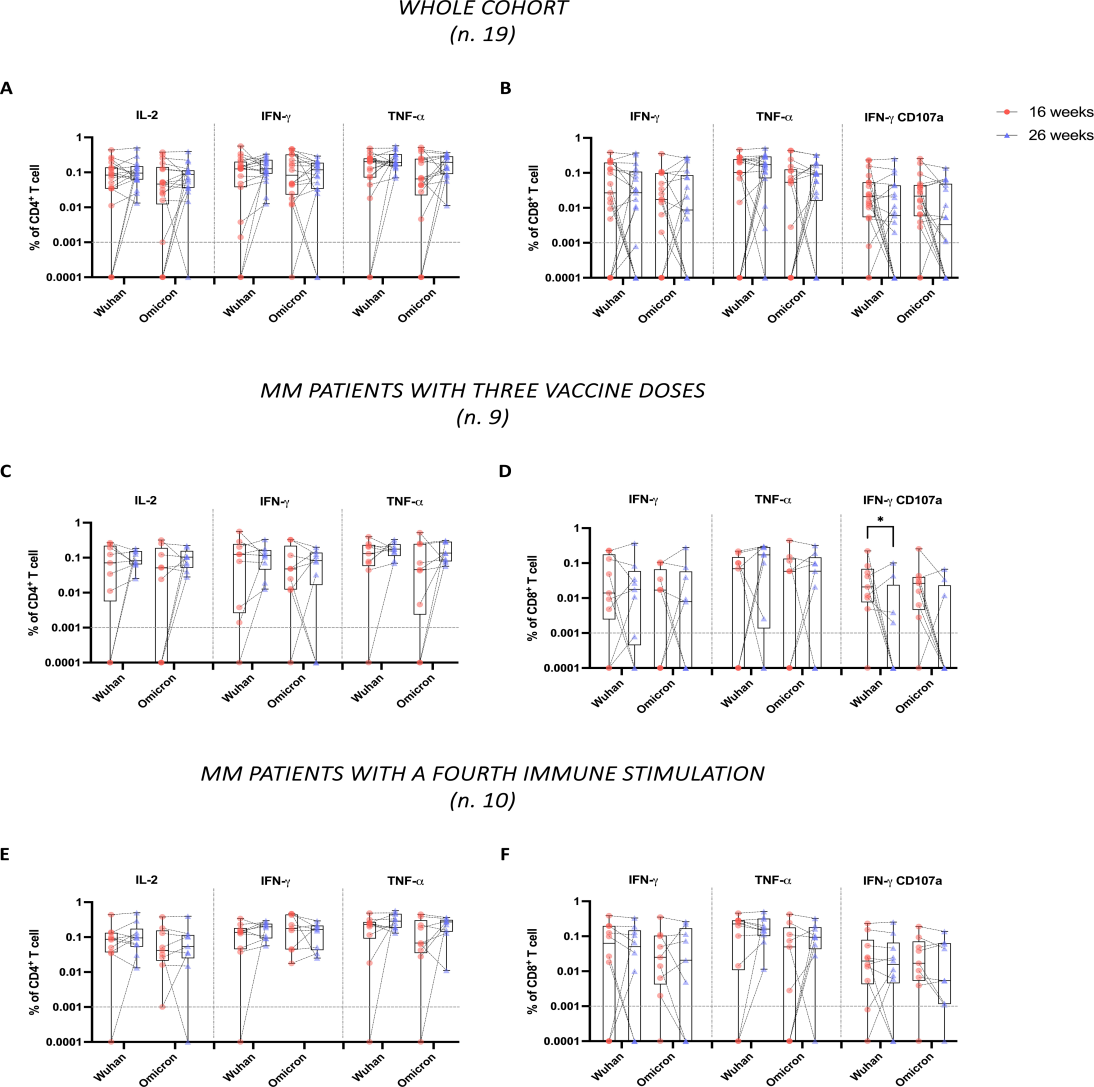
Stability of CD4^+^ and CD8^+^ T cell responses to Wuhan and Omicron spike proteins in multiple myeloma (MM) patients following third dose vaccine doses. Spike-specific T cells were evaluated by ICS flow cytometry analysis, following overnight stimulation of PBMCs with peptide pools covering the spike protein sequence from either the original Wuhan strain or the Omicron variant. Data are represented as percentages within total CD4^+^ or CD8^+^ T cells and in relation to single or dual expression of IL-2, IFN-γ, TNF-α, and CD107a. **(A, B)** show, respectively, the percentages of CD4^+^ and CD8^+^ specific to both Wuhan and Omicron spike proteins across the whole cohort (n.19). **(C, D)** show the same analysis conducted in the subgroup of MM patients with only three vaccine doses (n.9), while **(E, F)** show the same analysis in the subgroup of MM patients with additional immune fourth stimulation between the two time points (n.10). In **(A–F)**, the limit of detection (<0.001%) is indicated by the horizontal dotted line. Individual data points are shown, lines connect paired samples, and bars show the median with IQRs. Statistical analyses were performed using a non-parametric Wilcoxon test. *P* values are shown when *P* < 0.05 (*P < 0.05).

To address this, we evaluated spike-specific T cell responses to both Wuhan and Omicron variants at 16 and 26 weeks following the administration of the third vaccine dose in 19 of the 29 patients for whom sufficient PBMCs recovery was achieved. Using ICS flow cytometry, we analyzed cytokine production in CD4^+^ and CD8^+^ T cells, specifically focusing on IL-2^+^, IFN-γ^+^, and TNF-α^+^ single cytokine-producing CD4^+^ T cells and IFN-γ^+^, TNF-α^+^, and double-positive IFN-γ^+^CD107a^+^ cytotoxic CD8^+^ T cells. Despite high variability among patients, the analysis in the whole cohort revealed no significant differences over time in the percentages of both CD4^+^ and CD8^+^ T cells specific to Wuhan and Omicron spike proteins (**Figures 3A, B**). However, a trend towards a decrease in the percentage of IFN-γ^+^CD107a^+^ CD8^+^ T cells was observed at 26 weeks, in response to both the Wuhan and Omicron spike proteins (*P* = 0.11 and *P* = 0.11, respectively) (**Figure 3B**).

Upon further investigation based on the number of immune stimulations, we once again observed no significant differences in the percentages of CD4^+^ T cell responses over time (**Figures 3C, E**). On the contrary, among CD8^+^ T cells, we observed that patients who received the three vaccine doses showed a significant reduction (*P* = 0.04) in IFN-γ^+^CD107a^+^ CD8^+^ T cells specific to the Wuhan spike (**Figure 3D**). Specifically, the number of these patients with detectable IFN-γ^+^CD107a^+^ CD8^+^ T cell responses decreased from 8 out of 9 at 16 weeks, to 4 out of 9 at 26 weeks. In parallel, we reported a trend in the reduction of IFN-γ^+^CD107a^+^ CD8^+^ T cell responses specific to the Omicron spike but did not reach statistical significance (*P* = 0.06), even if the number of patients with detectable IFN-γ^+^CD107a^+^ CD8^+^ T cell responses to the Omicron spike decreased from 8 out of 9 at 16 weeks to 3 out of 9 at 26 weeks (**Figure 3D**).

Conversely, patients who received four stimulations maintained stable levels of both CD4^+^ and CD8^+^ T cells specific to Wuhan and Omicron spike proteins (**Figures 3E, F**), with no decline in IFN-γ^+^CD107a^+^ CD8^+^ T cells.

Age-based stratification of the patient cohort revealed significant differences in CD4^+^ T cell responses. Specifically, patients aged ≥77 years exhibited a lower frequency of Wuhan-specific IFN-γ^+^ and TNF-α^+^ CD4^+^ T cells at 26 weeks (**Supplementary Figure 3Ai**). In contrast, no significant differences were observed for Omicron-specific CD4⁺ and CD8⁺ T cell responses across age groups (**Supplementary Figures 3Bi, 3Ci, 3Di**). However, steroid-treated patients showed significantly reduced percentages of IFN-γ^+^ CD8^+^ T cells and double-positive IFN-γ^+^CD107a^+^ CD8^+^ T cells at 26 weeks in response to both Wuhan and Omicron spike proteins compared to steroid-untreated patients (**Supplementary Figures 3Bii, 3Dii**). No significant differences were observed when stratifying by disease status or by the use of immunomodulatory treatments (data not shown).

Overall, these findings suggest a substantial stability of spike-specific cellular responses over time across the cohort. Nonetheless, the cytotoxic profile of spike-specific CD8^+^ T cell responses appears to be waning, potentially exacerbated by steroid therapy. Additional immune stimulations could help preserve full cytotoxic functionality.

### 3.4 Monitoring of breakthrough infections after the administration of the fourth vaccine dose in MM patients

During the study, a fourth SARS-CoV-2 vaccine dose became available for MM patients. On the date of introducing this fourth vaccine dose, 25 patients in our cohort were eligible to receive this additional immune stimulation. As previously described, 7 patients underwent the fourth vaccine dose between the two PB collection time points, and another 7 patients chose to receive this booster after the 26-week. Overall, 14 out of 25 patients were vaccinated with four doses, while 11 out of 25 remained vaccinated with three doses. During the follow-up period, which extended until the end of September 2022, none of the 14 patients (0%) who received four doses developed a breakthrough infection. In contrast, 5 out of 11 patients (45.4%) who did not receive the fourth dose experienced a proven infection.

These findings highlight the key role of the fourth dose in bolstering immunity against both the original SARS-CoV-2 strain and dominant variants, particularly in immunocompromised individuals such as those with MMD or MMR.

## 4 Discussion

The stability of the immune responses over time following each vaccination is a crucial issue in developing tailored vaccination and prevention strategies against SARS-CoV-2 infection in MM patients. As previously reported, these patients exhibit profound impairments in both humoral and cellular immunity, rendering them particularly vulnerable to infections, including SARS-CoV-2 (2, 3). Moreover, the combination of the disease’s inherent immunodeficiency and the effects of immunosuppressive therapies contributes to suboptimal immune responses to SARS-CoV-2 vaccination (4). Multiple studies have demonstrated inconsistent seroconversion rates and diminished nAb titers, especially in MM patients undergoing active treatment (10–18). However, accumulating data indicate that additional vaccine doses can significantly improve immune responses, particularly regarding humoral immunity (18–20). Notably, while additional booster doses, such as a third mRNA vaccine dose, significantly increase nAb titers, the response to immune-evasive variants, in particular Omicron subvariants, remains compromised (29).

Currently, limited studies assess the stability of immune responses in MM patients following the administration of a third dose of mRNA SARS-CoV-2 vaccines. The available studies are typically constrained to short observation periods, or they primarily address humoral immunity while ignoring the role of cellular immunity and the impact of VoC, such as Omicron, which exhibit significant immune evasion properties (19–22). These limitations underscore the importance of a continuous, comprehensive, and detailed investigation of immune response stability.

Considering these observations, our study aimed to evaluate both humoral and cellular immune responses at two time points, from 16 to 26 weeks after the third dose of mRNA vaccines, with a follow-up period extending to 36 weeks. Furthermore, we investigated the impact of a fourth immune stimulation, whether through breakthrough infection or an additional vaccine dose, with a particular emphasis on the stability of immune responses. Our study also included the analysis of nAb titers against both the Wuhan original strain and the Omicron variant spike proteins, along with specific cellular responses, thereby providing a comprehensive evaluation of the vaccine’s impact on this cohort.

Our results confirm the evidence that the third vaccine elicits a robust humoral response, with 96.5% of patients seropositive for spike-IgG antibodies at 16 weeks post-vaccination. However, we observed a significant decline in spike-IgG antibody levels between 16 and 26 weeks in patients who did not receive additional immune stimulation. This waning of humoral immunity is consistent with results described in other immunocompromised populations, such as elderly care home residents and patients with other hematological malignancies (22, 27, 28). In contrast, patients who received a fourth stimulation maintained stable antibody levels, emphasizing the critical role of continued immune stimulation in sustaining strong responses in MM patients.

The analysis of nAb titers yielded comparable results. MM patients who received only three doses of the vaccine exhibited a statistically significant reduction in nAb titers, against both the Wuhan and Omicron spike proteins. It is noteworthy that the fourth immune stimulation resulted in a notable increase in nAb titers against both spikes, aligning with previous data that underscore the importance of timely administration of booster doses in sustaining cross-variant immunity within this high-risk population and potentially reducing the risk of breakthrough infections (29).

The decline in humoral and cellular immunity observed in MM patients following three doses of the SARS-CoV-2 vaccine highlights the challenges of achieving long-lasting immune protection in this vulnerable population. These observations are also supported by recent insights into immune imprinting, where prior vaccinations or infections shape the immune system’s response to subsequent boosters, potentially limiting the development of broadly nAbs in MM patients. Hybrid immunity, achieved through controlled additional exposures, could therefore enhance immune durability and cross-variant recognition, especially against VoC.

However, recent data indicate that while bivalent boosters broaden antibody specificity, they may not sufficiently enhance nAb responses to emerging Omicron subvariants, such as XBB.1.5 (32). These limitations underscore the need for repeated booster doses to sustain effective immunity in MM patients and suggest that future vaccine strategies may require personalization to overcome immune evasion by emerging variants.

In our cohort, we sought to evaluate potential confounding factors influencing the humoral immune response. No significant differences in spike-IgG antibody levels or nAb titers were observed based on the type of mRNA vaccine (mRNA-1273 or BNT162b2) administered as the third dose. Similarly, among patients who received the three-dose regimen, age, disease status, or concurrent therapy with steroids or iMDs did not significantly impact humoral responses at either of the studied time points.

Notably, among patients who received a fourth immune stimulation, significantly lower responses at 26 weeks were observed in patients aged ≥77 years and those with MMR compared to their younger counterparts and those with MMD, respectively. These findings underscore the negative impact of advanced age and disease status on the immunogenicity of booster doses, further supporting the need for tailored vaccination strategies in this vulnerable population.

Concerning the cellular response, we found substantial stability in spike-specific T cell responses, suggesting that cellular immunity may be more long-lasting than humoral immunity. However, a reduction in a subset of spike-specific CD8^+^ T cell responses, specifically in double-positive IFN-γ^+^CD107a^+^ T cells, was noted between 16 and 26 weeks in patients who did not receive further immune stimulation. The specific reduction in double-positive IFN-γ^+^CD107a^+^ CD8^+^ T cells might be related to differences in the timing between the production of cytokines and the storage of cytotoxic granules within these circulating CD8^+^ T cells. Another hypothesis is that these T cells exhibit a peculiar homing behavior; for instance, spike-specific IFN-γ^+^CD107a^+^ memory T cells might be predominantly tissue-resident rather than circulating in PB. Although this decrease was less pronounced than the reduction in humoral immunity, it may have clinical implications, given the role of cytotoxic T cell responses in controlling viral replication and preventing severe disease, particularly in the context of VoC with partial antibody escape (29). Interestingly, in this study, among patients who received a fourth vaccine dose or experienced a breakthrough infection, cytotoxic T cell responses remained stable, indicating once again that booster doses may be essential for preserving cellular immune function in this population (28).

The analysis of potential confounders revealed that patients aged ≥77 years had significantly lower percentages of Wuhan-specific CD4^+^ T cells producing IFN-γ and TNF-α at 26 weeks. Moreover, steroid therapy was associated with a significant reduction in IFN-γ⁺ and IFN-γ⁺CD107a⁺ CD8⁺ T cells, highlighting the detrimental impact of older age and immunosuppressive therapies on cellular immunity.

The clinical relevance of these findings is further highlighted by the observed infection rates at 32 weeks following the third vaccine dose. Almost half of the patients who did not receive a fourth vaccine dose experienced a breakthrough infection during the follow-up period, whereas no infections were reported in the group that received the additional dose. These findings indicate that regular monitoring and tailored vaccination strategies, with subsequent booster doses, may be crucial for maintaining adequate protection in MM patients, particularly those with relapsed or refractory disease who are at an elevated risk for severe outcomes.

While our study provides valuable insights into the stability of anti-SARS-CoV-2 immune responses in MM patients, it is not without limitations. The relatively small cohort size allowed for stratification based on variables such as age, disease status, and ongoing treatments; however, the statistical power of these analyses is inherently limited. Future studies with larger cohorts are necessary to validate these findings and provide more definitive conclusions. Nevertheless, the comprehensive assessments conducted at various time points provide a basis for elucidating the stability of immune responses in this population. Another limitation is that our analysis focused on the Omicron BA.1 variant, without considering different Omicron subvariants. Finally, the occurrence of asymptomatic breakthrough infections cannot be ruled out during the study conduction.

In conclusion, following a third dose of mRNA-based vaccine dose against SARS-CoV-2, both humoral and cellular responses decline over time in MM patients. A fourth stimulation seems crucial for the maintenance of immunity, particularly in the context of evolving SARS-CoV-2 variants, such as Omicron, and for the protection of the breakthrough infection risk. These results underscore the importance of continuous monitoring and optimizing vaccination strategies, potentially with variant-specific or CD8^+^ T cell-targeted vaccines, to protect MM patients from severe COVID-19 outcomes.

Overall, this study may help the benefit-risk evaluation of vaccination strategies in MM patients.

## Data Availability

The original contributions presented in the study are included in the article/**Supplementary Material**. Further inquiries can be directed to the corresponding authors.
