# Supplementary material for: Follow-up of humoral and cellular immune responses after the third SARS-CoV-2 vaccine dose in multiple myeloma patients
Source: Front Immunol. 2025 Feb 18;16:1532947. doi: 10.3389/fimmu.2025.1532947 (PMC11876378; doi:10.3389/fimmu.2025.1532947)
Supplement: Supplementary file 1 [file DataSheet1.pdf]

Supplementary Material

1 Supplementary Figures and Tables

1.1 Supplementary Figures

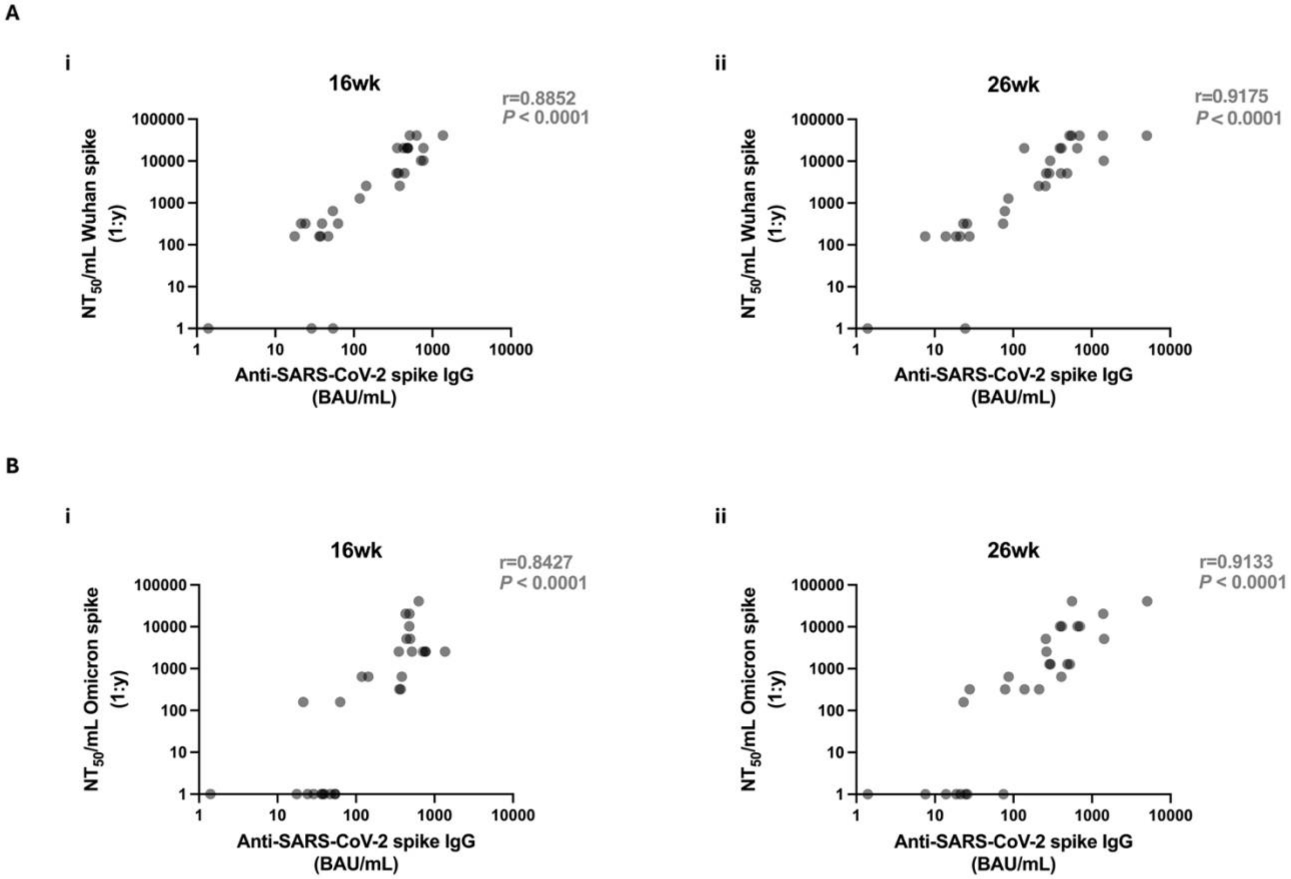

Supplementary Figure 1. Correlation between anti-SARS-CoV-2 spike IgG levels and neutralizing antibody titers against Wuhan and Omicron variants at 16 and 26 weeks post-vaccination. Scatterplots show the relationship between anti-SARS-CoV-2 spike IgG levels (measured in binding antibody units per milliliter) and neutralizing antibody titers (measured as the titer required to achieve 50% inhibition) against the Wuhan (A) and Omicron spike protein (B) at 16 (i) and 26 weeks (ii) for the entire cohort (represented by gray dots). Spearman's correlation coefficients and corresponding *P*-values are shown for each panel.

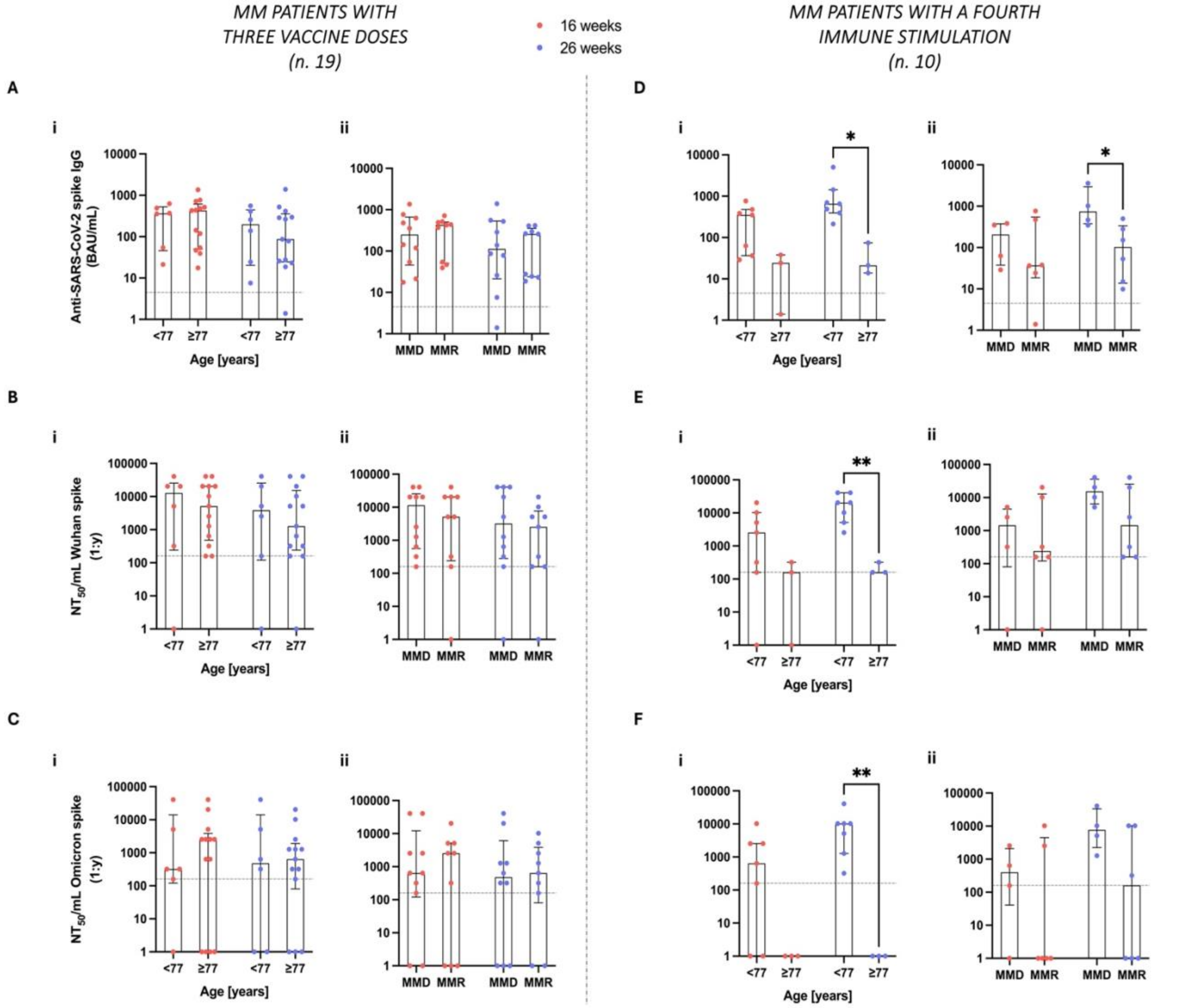

Supplementary Figure 2. Humoral responses to SARS-CoV-2 vaccination stratified by age and disease status in patients with multiple myeloma (MM). Panels (A–C) represent humoral responses in patients who received three vaccine doses (n = 19), while panels (D–F) show responses in patients who underwent a fourth immune stimulation through an additional vaccine dose or breakthrough infection (n = 10). (A, D) Anti-SARS-CoV-2 spike IgG levels (binding antibody units per milliliter [BAU/mL]); (B, E) neutralizing antibody titers (NT_{50}/mL) against the Wuhan spike; and (C, F) NT_{50}/mL against the Omicron spike. Data are stratified by age (<77 years vs. ≥77 years; i) or disease status (newly diagnosed MM [MMD] vs. relapsed/refractory MM [MMR]; ii). Responses were evaluated at 16 weeks (red symbols) and 26 weeks (blue symbols) post-vaccination. Individual data points are shown, and bars indicate median values with interquartile ranges. The limit of detection is indicated by the horizontal dotted line. Statistical analyses were performed using the unpaired, two-tailed, non-parametric Mann-Whitney U tests for comparisons between groups, with P values reported when $P < 0.05$ (*$P < 0.05$, **$P < 0.01$).

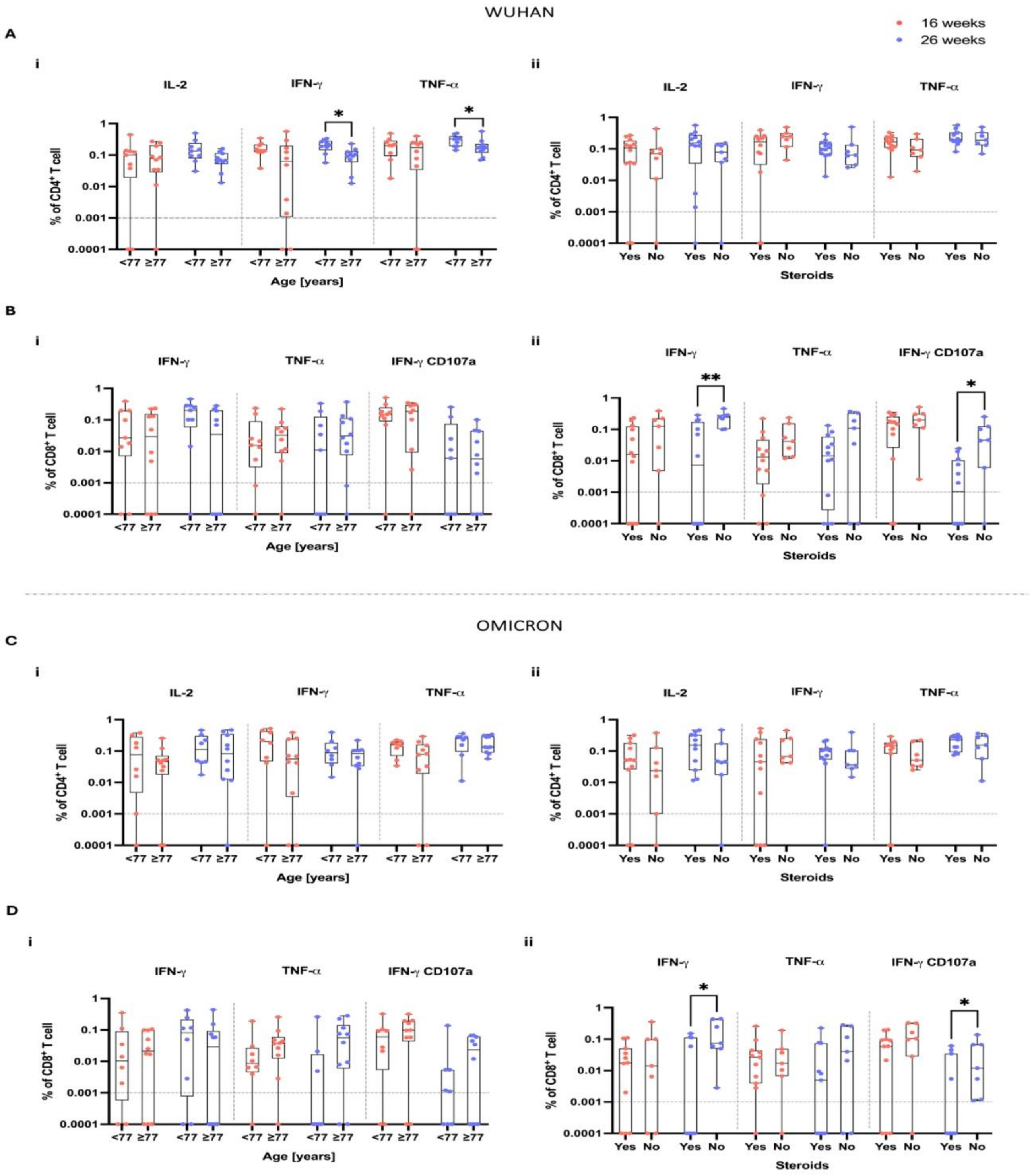

Supplementary Figure 3. Cellular immune responses to SARS-CoV-2 vaccination stratified by age and steroid therapy in patients with multiple myeloma (MM). Spike-specific T cells were assessed by intracellular cytokine staining flow cytometry following overnight stimulation of peripheral blood mononuclear cells with peptide pools spanning the spike protein sequences from either the original Wuhan strain or the Omicron variant. Data are presented as percentages within total CD4$^+$ or CD8$^+$ T cells, with analyses focusing on single or dual expression of IL-2, IFN-γ, TNF-

α, and CD107a. (A) and (B) depict the percentages of $CD4^+$ and $CD8^+$ T cells specific to the Wuhan spike, respectively, across the total cohort (n = 19), stratified by age (<77 vs. ≥77 years, i) or steroid use (Yes vs. No, ii). (C) and (D) show the same analyses for $CD4^+$ and $CD8^+$ T cells specific to the Omicron spike. The limit of detection (<0.001%) is indicated by horizontal dotted lines. Individual data points are displayed, with bars indicating median values with interquartile ranges. Statistical analyses were performed using the non-parametric Mann–Whitney U test for comparisons between groups. *P* values are reported when statistically significant (**P* < 0.05, ***P* < 0.01).

1.2 Supplementary Tables

Supplementary Table 1. Clinical characteristics of multiple myeloma patients.

	**MMD (n=14)**		**MMR (n=15)**	
Age (year)	77	[51-86]	77	[64-83]
Female gender	28.6%	(4)	60.0%	(9)
PCs in bone marrow biopsy	25.0%	[2-90]	40.0%	[5-98]
Lymphocyte Absolute counts ($x10^3/\mu L$)	1.46	[0.60-3.58]	1.04	[0.48-2.97]
**Immunoglobulin subtype**				
IgG	50.0%	(7)	46.7%	(7)
IgA	35.7%	(5)	20.0%	(3)
IgD	0.0%	(0)	0.0%	(0)
Non-secretory	0.0%	(0)	6.7%	(1)
Micromolecular	7.1%	(1)	26.7%	(4)
BJ	7.1%	(1)	0.0%	(0)
**Light chain isotype**				
κ	42.9%	(6)	60.0%	(9)
λ	57.1%	(8)	40.0%	(6)
**Cytogenetic risk**				
High	7.1%	(1)	13.3%	(2)
Standard	85.7%	(12)	66.7%	(10)
N/A	7.1%	(1)	20.0%	(3)
**Immunoparesis**				
None	14.3%	(2)	13.3%	(2)
1 immunoglobulin class	50.0%	(7)	6.7%	(1)
2-3 immunoglobulin classes	35.7%	(5)	80.0%	(12)

**ISS**				
I	28.6%	(4)	46.7%	(7)
II	50.0%	(7)	20.0%	(3)
III	21.4%	(3)	33.3%	(5)
**Previous line of therapy (n°)**	1	[1-2]	2	[2-6]
**Treatment regimen at first PB collection:**				
IMiDs	57.1%	(8)	80.0%	(12)
PI	28.6%	(4)	20.0%	(3)
Steroids	35.7%	(5)	80.0%	(12)
Anti-CD38 mAb	14.3%	(2)	26.7%	(4)
No active tratment	14.3%	(2)	0.0%	(0)

Note: values are presented as percentage (n) or median [range];

High Cytogenetic Risk definition: presence of one or more of these cytogenetic alterations: t(4:14), t(14:16), t(14:20), del17p;

Abbreviations: MM: Multiple Myeloma; D: Newly Diagnosed; R: Relapsed; PC: plasma cell; BJ: Bence Jones; ISS: International Staging System; PB: Peripheral blood; IMiDs: Immunomodulatory Drugs; PI: Proteasome Inhibitors.
